# Entropic Forces and Newton’s Gravitation

**DOI:** 10.3390/e22030273

**Published:** 2020-02-27

**Authors:** Angelo Plastino, Mario Carlos Rocca

**Affiliations:** 1 Consejo Nacional de Investigaciones Científicas y Tecnológicas, (IFLP-CCT-CONICET)-C. C. 727, 1900 La Plata, Argentina; rocca@fisica.unlp.edu.ar; 2 Departamento de Física, Universidad Nacional de La Plata, 1900 La Plata, Argentina; 3 Departamento de Matemática, Universidad Nacional de La Plata, 1900 La Plata, Argentina

**Keywords:** gravitational entropic force, non-relativistic Schrödinger treatment, dark-matter’s origin

## Abstract

Our subject of interest here is entropic forces, as re-interpreted by Verlinde with reference to gravitation, that is, by appealing to Verlinde’s conception of an entropic (statistically emergent) gravity advanced in [Physica A 2018, 511, 139]. In a canonical ensemble framework, we will deal with a non relativistic quantum scenario. In it, we perform a non-relativistic Schrödinger treatment (ST) of gravity as an entropic force and are able to detect new kinds of bounded quantum gravitational states, not previously reported. These new bound states would provide us with a novel energy-source, not taken into account as yet. The present entropic force deviates from the Newton’s form only at extremely short distances. We propose, by specializing our results to gravitationally interacting bosons, a model for dark matter generation.

## 1. Introductory Materials

### 1.1. A First Quantization Procedure

We will here effect a “first quantization” procedure for Newton’s gravity (NG), inspired by the pioneering works of Gupta [1,2,3] and Feynman [4], who advanced a Quantum Field Theory (QFT) of Einstein Gravity (EG). By a first quantization of an interaction *V* one usually understand introducing *V* in a Schrödinger equation and solving it. Gupta-Feynman defined EG-quantum states using gravitons a mediators of the associated interaction. They faced two difficulties: EG’s QFT is neither re-normalizable nor unitary.

The aims here are much humbler: to establish a first-quantization procedure for Newton’s gravity by solving the Schrödinger equation associated to it via the concomitant potential V(r), that differs from the 1/r form at distances smaller than 1 micron.. This is done by obtaining V(r) using Verlinde’s hypothesis concerning gravity as an emergent statistical force derived from an entropic information measure, in a non-relativistic manner.

### 1.2. Gravitation as an Entropic Force

Verlinde advanced in 2011 a link between gravity and an entropic force [5]. The ensuing conjecture was later proved [6], in a classical scenario.

In Reference [5], gravity emerges as a consequence of information regarding the positions of material bodies, combining a thermal gravitation treatment to Hooft’s holographic principle. Accordingly, gravitation ought to be viewed as an emergent phenomenon. Such exciting Verlinde’s idea received a lot of attention. For instance, consult [7,8,9,10]. An outstanding review of the statistical mechanics of gravity is that of Padmanabhan’s [11].

Verlinde’s notions gave rise to works on cosmology, the dark energy hypothesis, cosmological acceleration, cosmological inflation, and loop quantum gravity. The pertinent literature is immense [8,9,10]. Remark on Guseo’s work [12]; he demonstrated that the local entropy function, related to a logistic distribution, is a catenary and vice versa, an invariance that can be conjoined with Verlinde’s conjecture regarding gravity’s emergent origin. Guseo puts forward a new interpretation of the local entropy in a system [12]. Verlinde describes gravity as an emergent phenomenon that springs from the quantum entanglement of small bits of space-time information [13,14].

Our path begins then with (1) the Gupta-Feynman suggestion of looking for quantum states of gravity. This search is implemented by (2) using Verlinde’s idea of gravity as an emergent statistical force, which leads then to (3) a gravitation interaction functional form V(r) that differs from Newton’s at distances smaller than 1 micron. This Verlinde functional form is (4) introduced as the potential term of a Schrödinger equation, that is (5) solved, so that its eigenstates become determined, (6) realizing Gupta-Feynman’s aspirations. We (7) analyze the eigenvalues and on such a basis make conjectures regarding dark matter. To repeat: we speak of the eigenvalues of Verlinde’s V(r), that differs from Newton’s potential near the origin.

### 1.3. Main Effects of a First Quantization of Entropic Gravity (EG)

EG, regarded by Verlinde as an emergent statistical force derived from an entropic information measure, differs at very short distances from the Newton’s form. The associated modified gravitation-potential, introduced into the Schrödinger equation, yields bounded quantified states [15]. These bound states would provide us with a new energy-source, not taken into account as yet. This was shown to happen for fermions in [15]. Just the energy of the associated ground state was seen to produce a sizeable amount of novel energy.

### 1.4. Present Goals

We will extend here the ideas of [15] to the realm of bosons. Verlinde’s entropic force, proportional to a gradient of the potential energy, is obtained from the entropic information measure. What information measure, precisely? That of a boson ideal gas, because we showed in a previous study [6] that the pertinent entropies produce a workable framework. In [6], the above mentioned process yielded a boson-boson gravitational potential V(r) that is proportional to 1/r for distances larger than 25 microns. For smaller distances new, more involved contributions emerge. Thus, the ensuing potential V(r) differs from the Newtonian one at very short distances.

We can then, and this is what we are going to do here, write down a Schrödringer equation (SE) for our boson-boson V(r) and solve the SE. The new contributions to the gravitation potential will generate unknown-until-now quantum gravitational boson-states. The pertinent bound states of this SE can be regarded as a new energy source to which no attention at all has been aid yet, as far as we know.

### 1.5. Structure of This Manuscript

In Section 2 we review those details of Reference [6] necessary for constructing the gravity potential V(r) to be employed in the present effort. We also show how to approximate V(r) in order to perform a quantum analytical treatment of it. We set $V\left(r\right)=\sum_{i=1}^{3}\;V_{i}\left(r\right)$. In our central argumentation, that of Section 3, we solve the Schrödinger equation (SE) separately for these three pieces. The treatment of $V_{1}\left(r\right)$ yields our most important new results. As just an example to illustrate on our treatment, we analyze in Section 4 a rather daring conjecture regarding dark matter. Rough numerical estimates are given. Finally, some conclusions are drawn in Section 5.

## 2. Quantifying a Boson-Boson Interaction

### 2.1. The Ensuing Potential Function

As stated above, the gravitation potential V(r) between two bosons of masses $m_{1}=m$ and $m_{2}=M$, respectively, for an N−boson gas, was derived in [6] via a micro-canonical treatment taken from [16]. Thus, we start our considerations with an already-established potential function to be inserted below into a Schrödinger equation (SE).

In this paper we will deal with identical bosons and, of course, m=M. Returning to [6], the information measure *S* for *N* bosons of total energy *K* was therein obtained. From it one deduces an entropic force $F_{e}$, that à la Verlinde, is associated to emerging gravity. The associated boson-boson potential V(r) of [6] will be discussed in this Section.

In deriving V(r) in [6] one defines two constants, *a* and *b*, for *N* bosons of total energy *K*, in the fashion (with $k_{B}$ Boltzmann’s constant, *e* Euler’s number and *h* Planck’s constant)

(1) $$a={\left(3N\right)}^{\frac{5}{2}}h^{3};\;b=32\pi {\left(\pi emK\right)}^{\frac{3}{2}},$$

together with the relation that defines the proportionality constant λ between $F_{e}$ and the entropic gradient [6] (*G* is gravitation’s constant)

(2) $$\lambda =8\pi GmM/3Nk_{B}.$$

It is then shown in [6] that V(r) acquires the form

$$V\left(r\right)=GmM\frac{b}{a}\left\{\frac{r^{2}}{2}\ln\left(1+\frac{a}{br^{3}}\right)-\frac{a^{\frac{2}{3}}}{2b^{\frac{2}{3}}}\left\{\frac{1}{2}\ln\left[\frac{{\left[r+{\left(\frac{a}{b}\right)}^{\frac{1}{3}}\right]}^{2}}{r^{2}-{\left(\frac{a}{b}\right)}^{\frac{1}{3}}r+{\left(\frac{a}{b}\right)}^{\frac{2}{3}}}\right]\right.\right.+$$

(3) $$\left.\left.\sqrt{3}\left[\frac{\pi }{2}-\arctan\left[\frac{2r-{\left(\frac{a}{b}\right)}^{\frac{1}{3}}}{\sqrt{3}{\left(\frac{a}{b}\right)}^{\frac{1}{3}}}\right]\right]\right\}\right\}.$$

Our job now is to dissect V(r).

### 2.2. A Taylor Approximation for V(r)

Schrödinger’s equation with such an awful potential is obviously not amenable to analytic treatment. Since here we are performing a first exploratory study, we need that kind of treatment, which motivates us to find a suitable approximation to V(r). We try an approach that consists in writing

(4) $$V\left(r\right)\approx V_{1}\left(r\right)+V_{2}\left(r\right)+V_{3}\left(r\right),$$

with $V_{1}$ the first order Taylor approach for *r* small enough. We do this for $0<r<r_{0}$, with $r_{0}=10^{-10}$m (a typical hydrogen atom’s length).

(5) $$V_{1}\left(r\right)=-\frac{\pi GmM}{\sqrt{3}}{\left(\frac{b}{a}\right)}^{\frac{1}{3}}H\left(r_{0}-r\right)=V_{0}H\left(r_{0}-r\right).$$

It is of the essence to realize that $V_{1}\left(r\right)$ in Equation (5) will support bound states if inserted into a Schrödinger equation. Their associated self-energies will provide an as-yet unaccounted-for energy source.

We will consider also a particular distance $r_{1}=25.0$ microns, an empirical figure [17], the minimum distance at which Newton’s force that has been verified to work. The pertinent approximation for large *r* has already been obtained in [6] (*H* is Heaviside’s function.

(6) $$V_{3}\left(r\right)=-\frac{GmM}{r}H\left(r-r_{1}\right).$$

For intermediate r−values, $r_{0}<r<r_{1}$ (as stated above, there is experimental evidence to choose $r_{1}=25$ micrometers [17]), we perform a harmonic interpolating-form, that we call W(r),between the two fixed distance values $r_{1}-r_{0}$. Thus, one writes

(7) $$V_{2}\left(r\right)=W\left(r\right)\propto r^{2}.$$

**Figure 1:**
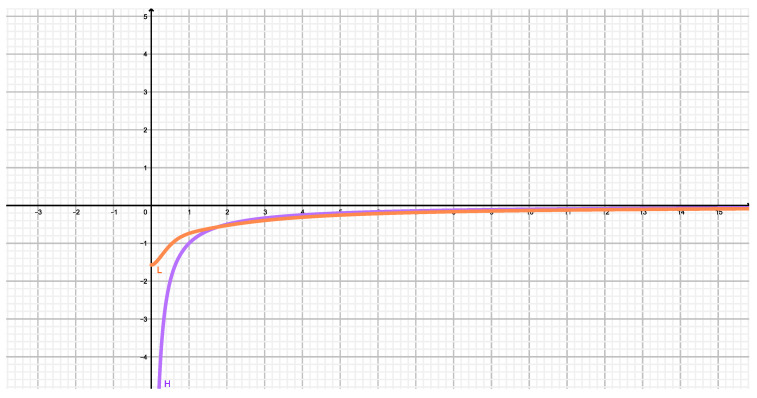
The orange curve (L) represents the exact potential given by (3) divided by $A=Gm^{2}/r_{2}$. It is finite at the origin! (no UV troubles at all then). The violet curve (H) represents the long-range (Newtonian) approximate potential $V_{3}/A$ given by (6), where the horizontal coordinate $x=r/r_{2}$ and $r_{2}={\left(a/b\right)}^{1/3}$.

Figure 1 compares our approximate potential long-range $V_{3}\left(r\right)$ to the exact one V(r) of Reference [6]. We can then see that the approximation is indeed quite good.

We pass now to a Schrödinger treatment (first quantization) of our approximate potential function $V_{1}+V_{2}+V_{3}$ for gravity.

## 3. Solving the Schrödinger Equation

We deal with

(8) $$U^{"}\left(r\right)+\left[-\frac{l(l+1)}{r^{2}}-\frac{2\mu_{r}}{\hbar^{2}}V\left(r\right)+\frac{2\mu_{r}}{\hbar^{2}}E\right]U\left(r\right)=0,$$

for

(9) $$V\left(r\right)\approx V_{1}\left(r\right)+V_{2}\left(r\right)+V_{3}\left(r\right).$$

We subdivide the treatment into three parts.

### 3.1. $V_{1}$ Discussion

For the first component, $V_{1}\left(r\right)$, essential for our present purposes, we have (see (5) for $V_{0}$)

(10) $$U_{1}^{"}\left(r\right)+\left[-\frac{l(l+1)}{r^{2}}+\frac{2\mu_{r}}{\hbar^{2}}\left(E-V_{0}\right)\right]U_{1}\left(r\right)=0.$$

Note that $l\left(l+1\right)\frac{h}{2\pi }$ is the eigenvalue of the angular momentum and $\mu_{r}$ the reduced mass of the two particles system. We define $s=\sqrt{\frac{8\mu_{r}\left(E-V_{0}\right)}{\hbar^{2}}}r$ and the associated solution reads

(11) $$U_{l1}\left(r\right)=A{\left(-is\right)}^{l+1}e^{-is}\phi \left(l+1,2l+2;-is\right)-B{\left(is\right)}^{l+1}e^{is}\phi \left(l+1,2l+2;is\right),$$

where ϕ stands for the hypergeometric confluent function [18]. Thus, the radial solution is

(12) $$R_{l1}\left(r\right)=A{\left(-is\right)}^{l+1}\frac{e^{-is}}{r}\phi \left(l+1,2l+2;-is\right)-B{\left(is\right)}^{l+1}\frac{e^{is}}{r}\phi \left(l+1,2l+2;is\right).$$

Consulting [18] we see that

(13) $$\phi \left(l+1,2l+2;-is\right)=2^{2l+1}e^{-i\pi (l+\frac{1}{2})}\Gamma \left(l+\frac{3}{2}\right)s^{-\left(l+\frac{1}{2}\right)}e^{-i\frac{s}{2}}J_{l+\frac{1}{2}}\left(\frac{s}{2}\right),$$

(Γ(z) is the Euler’s gamma function) and then [18]

(14) $$R_{l1}\left(r\right)=2^{2l+1}\Gamma \left(l+\frac{3}{2}\right)\frac{s^{\frac{1}{2}}}{r}\left(Be^{\frac{3\pi il}{2}}e^{3is2}-Ae^{-\frac{3\pi il}{2}}e^{-3is2}\right)J_{l+\frac{1}{2}}\left(\frac{s}{2}\right),$$

where *A* and *B* are two arbitrary constants, and $J_{l+\frac{1}{2}}$ is the well-known Bessel function [18]. Note that $R_{l}$ must comply with $R_{l}\left(r_{0}\right)=0,R_{l}^{{}^{\prime }}\left(r_{0}\right)=0$. Let $s_{0}=\sqrt{\frac{8\mu_{r}\left(E-V_{0}\right)}{\hbar^{2}}}r_{0}$. Then, the two boundary conditions lead to the demand

(15) $$J_{l+\frac{1}{2}}\left(\frac{s_{0}}{2}\right)=0.$$

Accordingly, $s_{0}/2$ must be a zero of the Bessel function. This zero will be called $\chi_{l,n}$, i.e.,

(16) $$s_{0}=2\chi_{l,n}=\sqrt{\frac{8\mu_{r}\left(E-V_{0}\right)}{\hbar^{2}}}r_{0}.$$

From here we see that the eigenvalue *E* becomes a quantized energy

(17) $$E_{l,n}=\frac{\hbar^{2}}{2\mu_{r}}\frac{\chi_{l,n}^{2}}{r_{0}^{2}}+V_{0},$$

where the zeros of the Bessel function provide the quantization scheme. We will see below in Section 4 that $V_{0}$ is very small. Thus, given the numerical value of the Bessel zeroes [18], we verify that $E>|V_{0}|$. With the definitions (2.1) for *a* and *b*, and the considerations made below that equation, one has now $a^{\frac{1}{3}}={\left(3N\right)}^{\frac{5}{6}}h$, $b^{\frac{1}{3}}={\left(32\pi \right)}^{\frac{1}{3}}{\left(\pi emK\right)}^{\frac{1}{2}}$. We have already fixed *K* but not yet *N*. For *N* we have given above just an order of magnitude. It will be further assessed in Section 4.

### 3.2. $V_{2}$ Discussion

For the harmonic interpolating potential we have

(18) $$U_{2}^{"}\left(r\right)+\left[-\frac{l(l+1)}{r^{2}}+\frac{2\mu_{r}}{\hbar^{2}}\left(E-W\left(r\right)\right)\right]U_{2}\left(r\right)=0.$$

We have two arbitrary constants *A* and *B* exactly as above for $V_{1}$. Here our radial solutions and their derivatives must comply, together, with four boundary conditions

1. $R_{l2}\left(r_{0}\right)=0$,
2. $R_{l2}^{{}^{\prime }}\left(r_{0}\right)=0$,
3. $R_{l2}\left(r_{1}\right)=0$, and
4. $R_{l2}^{{}^{\prime }}\left(r_{1}\right)=0$.

Since we have only two arbitrary constants (*A*, *B*) at our disposal and the boundary conditions are four, we can only satisfy three of these four boundary conditions (remember normalization). The first two boundary conditions are satisfied using just one of the two arbitrary constants plus the energy. The third boundary condition forces the second arbitrary constant to vanish, and, as a consequence, we obtain $R_{l2}\left(r\right)=0$. The fourth boundary condition does not need to be used.

### 3.3. $V_{3}$ Discussion

$V_{3}$ is the Newton gravitation potential. Here we deal with

(19) $$U_{3}^{"}\left(r\right)+\left[-\frac{l(l+1)}{r^{2}}+\frac{2\mu_{r}}{\hbar^{2}}\left(E+\frac{GmM}{r}\right)\right]U_{3}\left(r\right)=0,$$

noting that Whitaker’s function *W* solves the differential equation

(20) $$W^{"}+\left(-\frac{1}{4}+\frac{\lambda }{z}+\frac{\frac{1}{4}-\mu^{2}}{z^{2}}\right)W=0.$$

#### 3.3.1. Analysis of the Case E<0

Define $\mu =l+\frac{1}{2}$ and $\lambda =\frac{GmM}{\hbar }\sqrt{\frac{\mu_{r}}{2|E|}}$, and $s=\sqrt{\frac{8\mu_{r}\left|E\right|}{\hbar^{2}}r}$. The solution to (19) becomes

(21) $$U_{3}\left(r\right)=AW_{\lambda ,\mu }\left(s\right)-BW_{-\lambda ,\mu }\left(-s\right),$$

where $W_{\lambda ,\mu }\left(z\right)$ is given by

$$W_{\lambda ,\mu }\left(z\right)=\frac{{\left(-1\right)}^{2\mu }z^{\mu +\frac{1}{2}}e^{-\frac{z}{2}}}{\Gamma \left(\frac{1}{2}-\mu -\lambda \right)\Gamma \left(\frac{1}{2}+\mu -\lambda \right)}\left\{\sum_{k=0}^{\infty }\frac{\Gamma \left(k+\mu -\lambda +\frac{1}{2}\right)}{k!(2\mu +k)!}\right.\times$$

$$\left[\psi \left(k+1\right)+\psi \left(2\mu +k+1\right)-\psi \left(\mu +k-\lambda +\frac{1}{2}\right)-\ln z\right]+$$

(22) $$\left.{\left(-z\right)}^{-2\mu }\sum_{k=0}^{2\mu -1}\frac{\Gamma \left(2\mu -k\right)\Gamma \left(k-\mu -\lambda +\frac{1}{2}\right)}{k!}{\left(-z\right)}^{k}\right\}$$

Here 2μ+1 is a natural number, the last sum above must vanish for μ=0 and $\psi \left(z\right)=\frac{d}{dz}\Gamma \left(z\right)$. Accordingly,

(23) $$R_{l3}\left(r\right)=r^{-1}\left[AW_{\lambda ,\mu }\left(s\right)-BW_{-\lambda ,\mu }\left(-s\right)\right].$$

The operating radial boundary conditions are now $R_{l3}\left(r_{1}\right)=R_{l3}^{{}^{\prime }}\left(r_{1}\right)=0$. They lead to

(24) $$W_{\lambda ,\mu }^{{}^{\prime }}\left(s_{1}\right)+\frac{W_{\lambda ,\mu }\left(s_{1}\right)}{W_{\lambda ,\mu }\left(-s_{1}\right)}W_{-\lambda ,\mu }^{{}^{\prime }}\left(-s_{1}\right)=0.$$

Let $\sigma_{l,n}$ be the zeroes of this equation. Then, $s_{1}$ is one of them.

(25) $$s_{1}=\sigma_{l,n}.$$

Note that, from experiment [17] we can set $r_{1}=25$ micrometers. As we have (reasoning as in (3.8)) $s_{1}=\sqrt{\frac{8\mu_{r}\left|E\right|}{\hbar^{2}}r_{1}}$, then the energy becomes quantized and given, according to the $s_{1}$ values, by

(26) $$E_{l,n}=-\frac{\hbar^{2}}{8\mu_{r}}\frac{\sigma_{l,n}^{2}}{r_{1}^{2}}.$$

For example, setting l=0 we have that the energy differences between two contiguous excited states is of the order of $10^{-19}$ Joules. The quantization is thus somewhat fictitious. There is an effectively continuous energy, as one should expect.

#### 3.3.2. Analysis of the Case E>0

In this instance we have $\mu =l+\frac{1}{2}$, $\lambda =-i\frac{GmM}{\hbar }\sqrt{\frac{\mu_{r}}{2E}}$, and $s=\sqrt{\frac{8\mu_{r}E}{\hbar^{2}}r}$. The ensuing solution is

(27) $$U_{3}\left(r\right)=AW_{\lambda ,\mu }\left(-is\right)-BW_{-\lambda ,\mu }\left(is\right),$$

and then

(28) $$R_{l3}\left(r\right)=r^{-1}\left[AW_{\lambda ,\mu }\left(-is\right)-BW_{-\lambda ,\mu }\left(is\right)\right].$$

Radial boundary conditions are again $R_{l3}\left(r_{1}\right)=R_{l3}^{{}^{\prime }}\left(r_{1}\right)=0$, that translate into

(29) $$W_{\lambda ,\mu }^{{}^{\prime }}\left(-is_{1}\right)+\frac{W_{\lambda ,\mu }\left(-is_{1}\right)}{W_{\lambda ,\mu }\left(is_{1}\right)}W_{-\lambda ,\mu }^{{}^{\prime }}\left(is_{1}\right)=0.$$

Let $\varsigma_{l,n}$ be the zeroes of this equation. Then,

(30) $$s_{1}=\varsigma_{l,1}.$$

The energy becomes quantized once again

(31) $$E_{l,n}=\frac{\hbar^{2}}{8\mu_{r}}\frac{\varsigma_{l,n}^{2}}{r_{1}^{2}}.$$

Again, setting l=0 we have that the energy differences between two contiguous excited states is of the order of $10^{-19}$ Joules. The quantization is thus somewhat fictitious. There is a continuous energy, as one should expect.

## 4. A Dark Matter (DM) Model

### 4.1. DM and Entropic Gravity

Theorists of entropic (emergent) gravity put forward that what has been regarded as unobserved dark matter might instead be the product of quantum effects that produce an emergent energy (EE) [13,19,20].

We proceed here to build a dark matter model by appeal to three hypothesis.

First hypothesis (1): this EE will might be attributed to the gravitational interaction between two bosons, as we discussed above.

An instantiation of bosons is axions. The axion is an as-yet undetected particle introduced by Peccei and Quinn in 1977 [21,22,23] and used to solve the strong CP problem in the Standard Model (SMo). As it is well-known, the charge conjugation and parity (CP) symmetry is violated by the weak interaction in the SMo. The same does not happen with the strong interaction, which poses a dilemma. The solution was found by Peccei and Quinn by hypothesizing a new particle, the axion (see article by Frank Wilczek [24]). With it, the problem of the conservation of CP symmetry in the strong interaction could be solved. It has a very small mass, roughly 1.25 milli electron volts [25]. A posteriori, the axion was postulated as a candidate for solving the problem of dark matter [26]. Summing up, the axion is a hypothetical elementary particle. Should it exist, it might be regarded as a possible component of cold dark matter.

Second hypothesis (2): It is further assumed that axions interact amongst themselves only via gravitation (remember that ours is just an exploratory model).

Third hypothesis (3): Consider now an ideal gas of (mutually) gravitationally-interacting axions The number of axions is very great, of the order of $N=10^{79}$ (see below). *K* is assumed here to be the amount of energy equivalent to the total dark matter mass in the observable universe, estimated as $K=2.96\times 10^{84}$ eV [27].

### 4.2. Our DM-Model: Rough Numerical Estimates

We put together some numerical values now, and extract some important numerical values.

- (1) Calling $m_{a}$ to the axion mass, we have $m_{a}=1.25$ milli electron volt [25].
- (2) We saw above that the energy equivalent of the total dark mass in the observable Universe is $K=2.96\times 10^{84}$ eV [27].
- (3) We verify now that, indeed, $|V_{0}|$ is very small, as had been anticipated in Sect. 3 above. Its magnitude is $\frac{8.7}{N^{\frac{5}{6}}}\times 10^{-23}$ eV Thus (setting N=1), we have $|V_{0}|<9.8\times 10^{-22}$ eV, which is negligible compared to the first sum that appears in (17), where we have for the ground state $E_{0,0}$ a value $\chi_{0,0}=\pi$ and $r_{0}=10^{-10}$. Thus, we immediately get $E_{0,0}=9.75\times 10^{4}$ eV.
- (4) Therefore the number *N* of axions in the observable universe becomes approximately $2K/E_{0,0}∼6\times 10^{79}$, if, as assumed here, the energy $E_{0,0}$ would be the sole origin of dark matter.

## 5. Summary

- Our path began with the Gupta-Feynman suggestion of looking for quantum states of gravity.
- This search was implemented by using Verlinde’s idea of gravity as an emergent statistical force, which lead then to
- a gravitation interaction functional form that differs from Newton’s for distances smaller than 25 microns.
- This Verlinde functional form was introduced as the potential term of a Schrödinger equation.
- the equation was solved, so that its eigenstates became determined,
- thus realizing Gupta-Feynman’s aspirations.
- We analyzed the pertinent eigenvalues and on such a basis made conjectures regarding dark matter.

Accordingly, the logic of this paper has been as follows.

1. We started by accepting Verlinde’s suggestion that gravitation emerges from an entropic information measure *S*.
2. For a gas of free bosons we calculated in [6] (1) *S*, (2) Verlinde’s entropic force $F_{e}$, and from it (3) the gravitation potential V(r). We also found in [6] that V(r) deviates from the Newton’s form only at extremely short distances.
3. We have approximated above V(r) in a suitable fashion so as to obtain analytical solutions for the Schrödinger equation of potential $V\left(r\right)=V_{1}+V_{2}+V_{3}$. It is of the essence to realize that $V_{1}\left(r\right)$ in Equation (5) supports bound states of the Schrödinger equation. Their associated self-energies provides then an as-yet unaccounted-for energy source.
4. To repeat, the novelty of our treatment emerges at very short distances (the $V_{1}$ component of V(r)). The low-lying Schrödinger quantum states provide a novel energy-source, not accounted for previously. The pertinent energy eigenvalues yield, via Einstein’s relation energy $=mc^{2}$, a significant quantity of matter, that we might identify as dark one, of the order of five times the extant quantity of ordinary matter. As a matter of fact, one can limit oneself to the energy of the ground state of our Schrödinger equation to account for the extant amount of dark matter in the observable Universe.
5. As just an illustration of the above line of reasoning, we considered a hypothetical dark matter model based on three hypotheses. The model involves a conjectural dark matter generating mechanism, working through (mutually) gravitationally interacting axions, in which the entropic (2-body) gravity potential according to Verlinde, emerges from a gas of axions.

Equation (3), after suitably approximating it, yields analytical solutions to a concomitant Schrödinger equation. With its solutions we obtain a large quantity of quantized gravitational energy. Rough numerical estimates provide an arguably substantial amount of unobserved energy that, in Verlinde’s spirit, could be regarded as dark matter. Indeed, we can accommodate things so that our model’s mechanism might account for a large fraction of the extant dark matter. Let us insist that these results are putative ones, to be taken with a grain of salt. How effective is the present mechanism for dark matter generation is left for subsequent research. The mechanism is simple enough and could perhaps supplement the amount of dark matter supplied by entanglement between bit of space-time, as discussed in [19,28,29,30,31,32,33].

The essence of our discourse is to be found is the small difference, near the origin, between Newtons’s potential force and its entropic counterpart. This difference accounts for new, quantized low-lying self-energies to which our model attributes dark matter’s origin.
